# Association of the availability of pharmaceutical facilities provided in secondary and tertiary hospitals with clinical pharmacists’ work performance

**DOI:** 10.1186/s12913-023-10390-1

**Published:** 2023-12-06

**Authors:** Rong Cai, Xiaoyu Xi, Yuankai Huang

**Affiliations:** China Pharmaceutical University School of International Pharmaceutical Business, No. 639, Longmian Avenue, Jiangning District, Nanjing, Jiangsu Province 211198 China

**Keywords:** The availability of facilities, Clinical pharmacists, Work performance

## Abstract

**Background:**

Clinical pharmacists always work as the pivotal role in the process of facilitating the proper use of drug. Based on the person-environment fit theory, the availability of facilities required in pharmaceutical service may influence pharmacists’ performance, but which of them may have positive or negative impact remains unclear.

**Objectives:**

This study aims to analysed the quantitative association of the availability of pharmaceutical facilities provided in Chinese hospitals and clinical pharmacists’ work performance to assist hospitals formulating plans of the improving pharmaceutical working conditions to enhance clinical pharmacists’ performance.

**Method:**

Demonstrated by the panel of expert and literature review, the questionnaire for administrators and clinical pharmacists of secondary and tertiary hospitals in China was formed. Then a mixed sampling was adopted to gather data on information of the participants, as well as evaluation indexes of the availability of facilities and clinical pharmacists’ work performance.

**Results:**

Overall, 625 questionnaires distributed to administrators of hospitals and 1219 ones distributed to clinical pharmacists were retrieved. As for the Pharmaceutical facilities, while the increased availability of Traditional Chinese medicine pharmacy (*p* = 0.02) has a significantly positive impact on clinical pharmacists’ performance, the great availability of the preparation room (*p* = 0.07) negatively influences their work performance.

**Conclusion:**

Improving the availability of facilities that significantly influence clinical pharmacists’ work performance possibly reduce their workload, enhance their efficiency and further promote progress in pharmaceutical service.

**Supplementary Information:**

The online version contains supplementary material available at 10.1186/s12913-023-10390-1.

## Introduction

Clinical pharmacists always work as the pivotal role in the process of facilitating the proper use of drug. Their work performance referring to the extent to which their work is carried out, which significantly influence the diagnosis and evaluation of the diseases, the proper design and implementation of drug treatment regimens, as well as the assessment and feedback of treatment outcomes [1, 2]. Although the current pharmaceutical service conditions in China’s hospitals have been increasingly improved, there are still problems such as clinical pharmacists’ work not receiving the attention and recognition of hospital administrators [3], and their status, position, and fundamental rights in hospitals not being guaranteed as they should be [4]. With the competency of clinical pharmacists in Chinese hospitals, the hospitals’ conditions to support their work seems to be defective, which may cause errors and omissions in prescription or dispensing [5], further bringing negative influence to pharmacists’ work performance.

As one of the classical sociological theories, person-environment fit theory has been widely applied to study how different roles behave to achieve their goals in particular conditions [6]. According to this theory, the adaptability between objective conditions supplied and individual demand in pharmaceutical service system means the extent of the objective working conditions supporting that clinical pharmacists actually assume their responsibilities [7]. The objective conditions for clinical pharmacists’ work mainly refer to pharmacy professionals’ workplace and the availability of required facilities [8–10]. The availability of facilities required in clinical pharmacists’ work would affect their performance [11], and limited availability can be one of the major obstacles for them to implement pharmaceutical services [12].

At present the relationship between the availability of facilities required in clinical pharmacists’ work and their performance has received scant attention. There have been researches reckoned that factors like the development of pharmacy’s construction possibly feature prominently when clinical pharmacists carry out their work [13–15]. A few studies around the world emphasize the availability of facilities like residential aged care facilities [16–18], long-term care facilities [19], correctional facilities [20], supportive living and assisted living memory care facilities [21] are important for clinical pharmacists to provide outstanding service for specific populations. In developed countries, advanced pharmaceutical facilities are of great attention. For example, the appropriate facilities and equipment to ensure a sterile product are necessary conditions for pharmacists to dispense complex compoundings [22]. In developing countries, the preparedness of basic pharmaceutical facilities like dispensing pharmacy, medicine storage [23] and emergency pharmacy [24] positively influences pharmacists’ work performance. However, so far, no studies have obtained concrete evidence to confirm what detailed facilities provided by medical intuitions might have a positive or negative impact on the performance of pharmacists in China. Moreover, it is difficult to reach a definitive conclusion with indirect evidence such as review of availability of facilities used in clinical pharmacists’ work. Without clarifying the impact of various facilities on the performance of clinical pharmacists, it is hard to reduce the workload of clinical pharmacists and improve their work motivation and efficiency in a targeted and focused manner, so as to improve the level of pharmacy services [25, 26]. In addition, current studies also have limitations such as research objects originating from hospitals at all levels causing the sample devoid of specificity [27–30], or all subjects being chosen from tertiary hospitals in first-tier cities with better economic development causing the sample lacking representativeness, and the sample size being too small [31, 32].

Applying the person-environment fit theory, this study analyses the adaptivity mechanism between objective conditions and demand of objective humans through conducting survey among wider sample. This study analyses the the quantitative relationship between the availability of pharmaceutical facilities provided by medical institutions and the work performance of clinical pharmacists through empirical research on administrators and clinical pharmacists in secondary and tertiary hospitals in China. It aims to assist hospitals formulate plans of the improving pharmaceutical working conditions to enhance clinical pharmacists’ performance.

## Method

### Study design

This was a cross-sectional survey of pharmacists, which was designed to collect the data of the availability of pharmaceutical facilities provided in secondary and tertiary hospitals as well as the work performance of clinical pharmacists. The health care system in China follows a 3-tier hierarchical structure, but this research only considered pharmacists from secondary and tertiary hospitals because clinical pharmacy services are mainly provided in the two level of hospitals, which account for more than half of the health care burden in China [25, 33]. Ordinary least squares regression was used to assess the association of each independent variable with clinical pharmacists’ work performance.

### Participants

The research was conducted from July to August in 2019. Involving surveyed participants were administrators and clinical pharmacists in Chinese hospitals. The inclusion criteria were as follows: first, the inclusion criteria of hospital administrators was (1) being appointed as the director or deputy director in charge of hospital pharmacy work or the section chief of the pharmacy department of the sampled hospitals; second, the inclusion criteria of clinical pharmacists were (1) working as full-time clinical pharmacists of the sampled hospitals; (2) undertaking specific duties involving management of pharmaceuticals, patients, or medical information. Clinical pharmacists in training (students on clerkships or internships) and visiting clinical pharmacists were excluded.

Moreover, these two groups of survey objects should be available and willing to participate in the study by completing the questionnaire that would take approximately 20–30 min. And they should be able to sign the informed consent document [34, 35].

A mixed sampling strategy was adopted. First, all 31 provincial administrative regions (including provinces, autonomous regions, and municipalities directly under the central government) in mainland China were covered in the sampling [36]. Next, cities in each province, autonomous region or district in each municipality were evenly divided into 3 groups according to their 2018 per capita gross domestic product, for this index was associated with the factors such as the quality of health care techniques and utilization of health care influencing the working ability and conditions of medical staffs. Thereby, 93 groups were generated. Within each group, 1 city or district was selected using the random number method; thus, 93 cities or districts were selected through the multi-staged sampling. In each selected city or district, 2 to 4 tertiary hospitals were surveyed by convenience based on the hospital administrators’ permission to conduct the survey. In each hospital surveyed, 2 hospital manager, and 4 clinical pharmacists were recommended by the hospital administrators who first contacted the investigator or other participants who completed the questionnaire. Overall, 372 hospitals would be sampled and a total of 744 hospital administrators and 1488 clinical pharmacist questionnaires were distributed in this study.

### Instrument

An expert panel of 2 administrators and 2 teaching clinical pharmacists from tertiary hospitals, together with 3 experts in clinical pharmacy education from universities, were consulted for the design of the study questionnaire. The questionnaire comprised 3 sections: information of the participants, the availability of pharmaceutical facilities, and clinical pharmacists’ work performance among which the second section would be filled by the hospital administrators and the third section would be filled by clinical pharmacists.

#### Information of the participants

Pharmacists’ gender, age, marital status, seniority, professional title, specialized field and the feature of hospitals were set as the covariant. These factors are potentially related to clinical pharmacists’ work performance based on literature studies [37].

#### Evaluation index of the availability of facilities required in pharmaceutical work

The required availability of pharmaceutical facilities in hospitals were summarized according to domestic related laws and regulations including Good Pharmacy Practice (GPP) and Regulations of Pharmaceutical Administration in medical institutions. Demonstrated by the panel of experts, evaluation index system of 11 items concerning facilities required in clinical pharmacists’ routine work was formed, which was presented to participants as “Has the hospital where you work been availed with the facilities in the following list” and with a response of “Yes” or “No”. According to the advice of the expert panel, clinical pharmacists probably be unable to provide the information about the availability of pharmaceutical facilities required for this study, while the administrators of medical institutions would be more familiar with this matter or be able to obtain this information more conveniently. Therefore, this part of the questionnaire was filled by medical work administrators.

#### Evaluation index of clinical pharmacists’ work performance

Existing instruments for evaluating the work achievement of specialists in pharmacy are only appropriate for pharmacists in social drugstore. Besides, their content is limited to the service that directly access to patients [38, 39]. They do not involve the assessment of the comprehensive responsibilities of clinical pharmacists. As a result, there was no existing suitable instrument for measuring the performance of clinical pharmacists in hospitals in China. Based on the practice guidelines for pharmacotherapy specialists issued by American College of Clinical Pharmacy (ACCP), guidelines on a standardized method for pharmaceutical care issued by American Society of Health-System Pharmacists (ASHP) and regulations on the Administration of Pharmaceutical Affairs in Chinese Medical Institutions together with literature review and expert panel interview, ultimately 12 items were concluded to represent all routine work of clinical pharmacists in Chinese hospitals. Each item was presented to participants as, “Have you ever undertaken the task?” with a response of a 5-level Likert scale, ranging from 1 (strongly disagree) to 5 (strongly agree). It was the total score that was included in regression analysis.

### Pre-test

All instruments were composed in Chinese or translated to Chinese and proofed by the expert panel and researchers, followed by a multi-round pilot survey in tertiary hospitals in Nanjing, Jiangsu province, China, to test their rationality, understandability, and readability. The pilot participants for each round met the general inclusion criteria for the study [36]. Five or six Participants were included in each pilot study. After each round, revisions involving polishing the expression of the questions to improve their readability were made according to the feedback. The pilot process continued until no new feedback was generated. Eventually, 4 rounds of pilot tests were completed. Because before the termination of the pilot study, there had been still some problems negatively influence the understandability of the questionnaire, the results received before were meaningless. Responses received during all pilot tests were not included in the final stage of data collection.

Subsequently, a pre-test of the questionnaire was conducted among 47 clinical pharmacists from 24 tertiary hospitals in 6 cities of Jiangsu province in China during April 2019 by convenience sampling. Reliability of the instrument for the clinical pharmacists’ work performance was acceptable (Cronbach’s alpha 0.63 for work performance instrument). The final questionnaire is available online as Additional file 1.

### Data collection

A total of 184 undergraduate students majoring in general pharmacy or clinical pharmacy were recruited as data collectors. They were trained to be able to access the potential participants and be familiar with the procedure of conducting the survey and the standardized explanations for potential questions from the participants. Every 2 data collectors investigated 1 set of geographically neighbouring cities or districts in pairs during July and August of 2019. After obtaining the hospital administrators’ consent, the data collectors asked the potential participants for their basic information to determine whether they meet the study inclusion criteria. Then they informed the eligible participants of the purposes, contents, and requirements of the survey and confirmed their willingness to participate again. Those who were willing to participate signed the consent form and decided the time and an undisturbed place for survey with the data collectors.

The data collectors orally interviewed the participants with each item of the questionnaire and recorded their responses through an online survey system installed on mobile phones or tablet computers. The online survey system would convert the data into electronic documents. The data collectors were not allowed to provide any view on the questionnaire, except the requirements or instructions of questionnaire filling. The survey system allowed the users to set restrictions on format of responses and ensured the quality of the data. A total of 5 postgraduates were recruited and trained to review the uploaded documents and immediately return those with data entry errors or damaged data, which were corrected through return visits by data collectors when possible [36].

### Data analysis

The dependent variable of this study was the work performance of clinical pharmacists. Because of the uncertainty of the 12 items’ devotion to the dependent variable, the following approach was adopted to determine the weight of each item, thereafter the weighted total score was used to represent the value of the dependent variable [40, 41]. (1) Conducting principal component factor analysis on 12 questions to obtain the principal component coefficients of each question. (2) If the variance contribution rate of the first principal component is relatively high, the final weight is obtained by normalizing this principal component coefficient. (3) Otherwise, the second and third principal components should be taken into account, each of which would be weighted based on the variance contribution rate. The coefficient of the principal component in each principal component will be divided by the corresponding characteristic root square and multiplied by this weight, and finally all the principal component coefficients of the question items will be normalized to get the final weight.

The main variable of the independent variables was the availability of facilities required in clinical pharmacists’ work, and the covariate included the gender, age, marital status, seniority, technical title, professional field and feature of hospitals of respondents. Descriptive statistics were used to report the characteristics of the sample. Ordinary least squares regression was used to assess the association of each independent variable with clinical pharmacists’ work performance. Multicollinearity was assessed by examining the variance inflation factor (VIF). An independent variable with a VIF value more than 10 means that it has collinearity within the other independent variables and should be removed. VIF was examined again when an independent variable with the highest VIF more than 10 was removed. This was repeated until multicollinearity was not suspected any more. Three levels of statistical significance were set in this study, namely *p* < 0.1, *p* < 0.05, *p* < 0.01. Stata 15.0 was used for data analysis.

## Result

Overall, 625 questionnaires distributed to hospital administrators were filled out completely (response rate = 84.00%). The feature of sampled hospitals was shown in Table 1.

**Table 1:** The feature of sampled hospitals

Types	Eastern N(%)	Western N(%)	Central N(%)	Total N(%)
General hospital	84 (26.9%)	73 (23.4%)	84 (26.9%)	241 (77.2%)
Specialized hospital	10 (3.2%)	1 (0.3%)	5 (1.6%)	16 (5.1%)
TCM hospital	23 (7.4%)	14 (4.5%)	12 (3.8%)	49 (15.7%)
Others	1 (0.3%)	1 (0.3%)	4 (1.3%)	6 (1.9%)
Total	118 (37.8%)	89 (28.5%)	105 (33.7%)	312 (100%)

Totally 1219 questionnaires distributed to clinical pharmacists were finally received (response rate = 81.48%). The main characteristics of participants are found in Table 2. The mean age of the participants was 35.72(SD = 7.16), and their mean years of practicing as a clinical pharmacist was 10.11(SD = 7.22). Approximately two-thirds of the participants were female (65.14%) and most were married (83.02%). More than half of the participants were with junior technical title (34.94%) or intermediate title (51.12%).

**Table 2:** Sociodemographic data of clinical pharmacists

Item	N (%)
Age (mean, sd)	35.72 (7.16)
Years of practice (mean, sd)	10.11 (7.22)
Gender, n (%)	
Male	425 (34.86%)
Female	794 (65.14%)
Marital status, n (%)	
Unmarried	198 (16.24%)
Married, n (%)	1012 (83.02%)
Others (divorced, widowed, etc.)	9 (0.74%)
Technical titles, n (%)	
Junior title	426 (34.94%)
Intermediate title	628 (51.52%)
Deputy senior title	139 (11.41%)
Senior title	26 (2.13%)
Education background, n (%)	
Lower than bachelor’s degree	95 (7.79%)
Bachelor’s degree	781 (64.06%)
Master’s degree	334 (27.39%)
Doctoral degree	9 (0.76%)

The availability of facilities in surveyed hospitals can be seen in Table 3. As for various facilities, outpatient pharmacies (100.0%), inpatient pharmacies (94.56%), pharmaceutical warehouse (98.24%), Chinese medicine clinics (94.24%) were generally available, which existed in almost all the surveyed hospitals. On the other hand, the data and information department (65.28%), the centralized intravenous drug dispensing room (50.56%), the preparation room (43.04%), the drug testing room (41.92%) and the pharmacy research room (29.76%) were limitedly available in Chinese hospitals.

**Table 3:** The availability of facilities

Item	N (%)
Outpatient pharmacy	625 (100%)
Emergency pharmacy	483 (77.28%)
Inpatient pharmacy	591 (94.56%)
Preparation room	269 (43.04%)
Laboratory of the control of drug	262 (41.92%)
Laboratory for research of drug	186 (29.76%)
Medicine store	614 (98.24%)
Clinical pharmacy	507 (81.12%)
Department of information	408 (65.28%)
Traditional Chinese medicine pharmacy	589 (94.24%)
Pharmacy of intravenous admixture service	316 (50.56%)

In Table3, laboratory of the control of drug is responsible for the inspection of semi-finished products, finished products, raw materials, and purchased drugs in the hospital, and is in charge of the quality management of drugs. As for substandard products, clinical pharmacists assist in analyzing the reasons for non-compliance by gaining a deeper understanding of the manufacturing process, and they will conduct technical reviews if necessary.

Clinical pharmacists in laboratory for research of drug conducts research and development work on new drugs, formulations, and dosage forms based on the medical, teaching, and scientific research needs within the hospital. In details, they conduct research on drug properties, dosage forms, drug quality, drug interactions, and compatibility contraindications, and they also should pay attention to pharmacokinetic, bioavailability, and toxicological studies.

Department of information is responsible for provide consultation information for clinical use. Staff here should master the development trends of domestic and international pharmacy, collecting and categorizing pharmaceutical information materials in time.

**Fig. 1:**
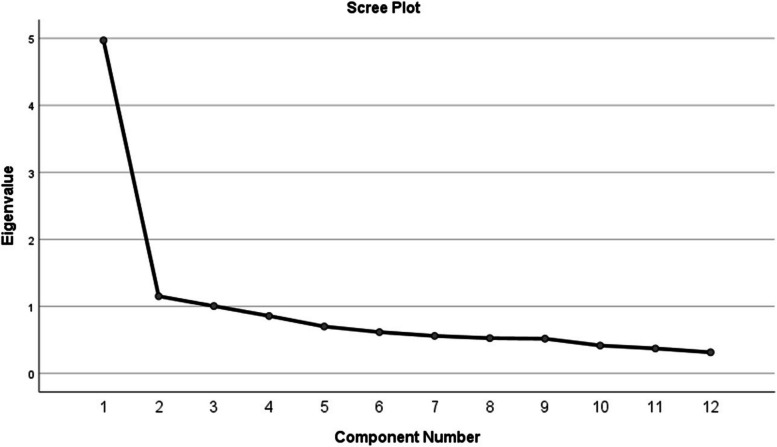
The scree plot of PCs

The results of principal component analysis of clinical pharmacists’ work performance scores were shown in Table 4. The KMO score was 0.889 and scree plot was shown in Fig. 1. The characteristic roots of the first three principal components were greater than 1, and the cumulative variance contribution rate was 59.37%. First, the coefficient of each question item in each principal component was divided by the sum of the corresponding principal component coefficients, and the absolute values of the results were calculated. Second, the percentages of variance of each principal were divided by the cumulative percentages of these principal components, so the variance’ contribution rate of each principal component were obtained. Thirdly, absolute values calculated in the first step were multiplied by the variance’ contribution rates correspondingly. The final weight corresponding to each question item was obtained after the results of the step three were divided by the sum of the them. And the comprehensive score of each sample is calculated accordingly.

**Table 4:** Weight of items to evaluate the clinical pharmacists’ work performance

Component	**Principal component**			Initial Eigenvalues			Extraction Sums of Squared Loadings			**Weight**
	1	2	3	Total	% of variance	cumulative %	Total	% of variance	cumulative %	
1	0.66	-0.41	0.088	4.97	41.413	41.413	4.97	41.413	41.413	0.085
2	0.636	-0.442	-0.072	1.151	9.593	51.006	1.151	9.593	51.006	0.084
3	0.589	-0.105	0.524	1.005	8.371	59.377	1.005	8.371	59.377	0.083
4	0.671	-0.274	0.15	0.856	7.136	66.513				0.083
5	0.55	-0.071	0.298	0.699	5.829	72.342				0.068
6	0.695	-0.05	-0.281	0.616	5.131	77.472				0.081
7	0.765	-0.098	-0.321	0.559	4.657	82.129				0.091
8	0.692	0.107	0.14	0.525	4.379	86.508				0.077
9	0.704	0.19	-0.205	0.518	4.316	90.823				0.084
10	0.636	0.255	-0.514	0.415	3.462	94.285				0.094
11	0.57	0.535	0.163	0.372	3.096	97.381				0.086
12	0.509	0.534	0.269	0.314	2.619	100				0.084
										1

The results of regression analysis was provided in Table 5. In case of facilities provided in hospitals, the traditional Chinese medicine pharmacy (Coef. = 0.22, *p* = 0.02, 95%CI = [0.03, 0.41]) has a significantly positive impact on the actual work of clinical pharmacists, the preparation room (Coef. = -0.13, *p* = 0.07, 95% CI = [-0.28, 0.01]) had a significantly negative impact on clinical pharmacists’ performance. Whether there is a drug researching department (Coef. = 0.1, *p* = 0.08, 95%CI = [-0.01, 0.21]) has certain significant correlation with the actual work carried out by the participants.

**Table 5:** Regression analysis of the availability of facilities’ association with clinical pharmacists’ work performance

Items	M1				M2			
Model *P* value	< 0.05				< 0.05			
*R*^2^	0.048							
Adjusted *R*^2^	0.046				0.046			
Items	Initial result				Robust result			
	Coef.	*P* >|t|	[95% Conf. Interval]		Coef.	*P* >|t|	[95% Conf. Interval]	
Outpatient pharmacy								
Emergency pharmacy	0.01	0.93	-0.10	0.11	0.01	0.94	-0.12	0.13
Inpatient pharmacy	0.00	0.98	-0.20	0.19	0.00	0.98	-0.19	0.18
Preparation room	-0.13	0.08*	-0.28	0.01	-0.13	0.07*	-0.28	0.01
Laboratory of the control of drug	0.07	0.38	-0.08	0.22	0.07	0.34	-0.07	0.20
Laboratory for research of drug	0.10	0.10	-0.02	0.22	0.10	0.08*	-0.01	0.21
Medicine store	-0.10	0.55	-0.41	0.22	-0.10	0.58	-0.43	0.24
Clinical pharmacy	0.05	0.44	-0.07	0.16	0.05	0.45	-0.08	0.17
Department of information	-0.02	0.73	-0.11	0.08	-0.02	0.74	-0.12	0.09
Traditional Chinese medicine pharmacy	0.22	0.02**	0.04	0.40	0.22	0.02**	0.03	0.41
Pharmacy of intravenous admixture service	0.06	0.28	-0.05	0.16	0.06	0.29	-0.05	0.16
Level of hospitals	0.06	0.19	-0.03	0.16	0.06	0.21	-0.04	0.16
Types of hospitals	0.01	0.52	-0.03	0.05	0.01	0.56	-0.03	0.06
Gender	-0.01	0.88	-0.09	0.08	-0.01	0.87	-0.09	0.08
Marital status	-0.01	0.86	-0.14	0.11	-0.01	0.85	-0.13	0.11
Technical title	0.03	0.41	-0.04	0.11	0.03	0.39	-0.04	0.10
Educational background	0.05	0.24	-0.03	0.12	0.05	0.21	-0.03	0.12
Age	0.00	0.42	-0.01	0.01	0.00	0.43	-0.01	0.01
Years of practice	0.00	0.82	-0.01	0.01	0.00	0.82	-0.01	0.01
Constant term	3.24	0.00	2.75	3.74	3.24	0.00	2.78	3.71

^*^P < 0.1

^**^P < 0.05

The result of the multiple regression model used in the study: y = 3.24 + 0.01x_1_ + 0.00x_2_ + (-0.13)x_3_ + 0.07x_4_ + 0.1x_5_ + (-0.1)x_6_ + 0.05x_7_ + (-0.02)x_8_ + 0.22x_9_ + 0.06x_10_ + 0.06x_11_ + 0.01x_12_ + (-0.01)x_13_ + (-0.01)x_14_ + 0.03x_15_ + 0.05x_16_ + 0.00x_17_ + 0.00x_18_ + ε.^1^

## Discussion

This study analysed the association of the availability of pharmaceutical facilities provided in hospitals with clinical pharmacists’ work performance. Multi-staged sampling was adopted to cover a wide range of sample so as to ensure it being highly representative. According to the results, among all facilities probably required in clinical pharmacists’ work, the availability of Traditional Chinese Medicine (TCM) pharmacy, the preparation room and drug researching room has significant impact on their work performance.

The reasons for the availability of the TCM pharmacy brings beneficial influence to clinical pharmacists’ performance may lies in two aspects. For one thing, it can play a positive role in case of the prescription consisted of both Chinese herbs and modern drugs. In such a circumstance, clinical pharmacists need to cooperate with other members of the medical team to perform their duties including assessment of patients’ drug demand, optimization of drug composition, and adjustment of drug dosage, etc. [42]. The combined treatment of traditional Chinese medicine and western medicine not only enhance the curative effect while reducing the adverse reaction but also increase intractability of administration of drug [43]. In general hospitals, it is common that patients consult more than one department to diagnose and treat different diseases, where doctors may write out complex prescriptions. Setting up the TCM pharmacy provides a platform for patients to conveniently consult the methods or precautions of taking decoction with Chinese patent medicine or taking traditional Chinese medicine with western medicine [44]. It is also beneficial for clinical pharmacists to learn about patients’ demand of drug comprehensively and further accurately evaluate the safety and effectiveness of the medication regimen [45]. Therefore, either adverse reactions caused by the interaction of Chinese herbs and modern drug or toxic side effect caused by overdose resulting from repeatedly administration can be effectively avoided. Apart from the patients, some physicians would like to turn to clinical pharmacists in TCM pharmacy to recognize the unfamiliar herb or uncomprehending point in prescriptions of traditional Chinese medicine which may be borrowed from other hospitals, so as to avoid incompatibility in practice [46]. Moreover, physicians may not master the proper use of rare and toxic Chinese medicines [47], and in such a circumstance, the TCM pharmacy provides a suitable place for clinical pharmacists to adjust the prescription dosage according to the individual conditions of patients. For another, the availability of TCM pharmacy makes it possible for clinical pharmacists to provide high-quality service like decocting herbal medicine [48], dispensing decocted pieces and preparing non-decocted granules [49] which can avoid the loss of drug efficacy caused by patients’ unscientific operation in the process of decocting as much as possible [50], thus improving the economy and rationality of drug treatment.

On the other hand, the availability of the preparation room has significant negative influence on clinical pharmacists’ work performance. It has been reported that since 2019, based on investigation on whether hospitals with drug approval numbers have established a preparation room [51], the facility has been removed from many hospitals. The reason for the phenomenon may lie in the fact that with the continuous improvement of the national standards for preparations of medical institutions, the limited size and obsolete equipment of hospitals’ preparation room hardly satisfy the requirements of manufacturing of drug and it was finally reduced to be closed [52]. Besides, with the increasing variety of drugs mushrooming on the domestic market, there are also cases where preparations of some medical institutions are gradually replaced by similar products sold on the market [53]. Inadequate facilities probably result in clinical pharmacists hardly striving for perfection at pharmaceutical preparation work constantly. The preparations made in medical institutions are directly related to clinical practice for they can be adjusted in time to meet clinical demand. Also, this practice can reduce the cost of circulation. And some of specific preparations are attached with definite curative efficacy, which have irreplaceable outstanding advantages [51]. Therefore, the medical institutions may be reminded to properly increase the investment in preparation room to expand its scale, standardize preparations’ production, provide good conditions for clinical pharmacists to carry out related work, promoting the innovative development of hospital preparations.

Outpatient pharmacy, inpatient pharmacy, etc. are common ancillary facilities needed in pharmaceutical service, which, however, have no significant impact on the work performance of clinical pharmacists in this study. Maybe because these facilities were introduced early in Chinese hospitals to develop considerable operational experience and have gradually applied artificial intelligence(AI) for automation transformation [54, 55]. Advanced technology helps to reduce pharmacists’ repeated work like delivering, supplementing and checking drug [56, 57], so they can devote themselves to pharmaceutical service demanding professional knowledge and skills such as prescription consultation in the TCM pharmacy and dispensing in the preparation room. Moreover, AI offers potential opportunities to optimize clinical pharmacy services in community or hospital settings. AI powered apps and tools currently play a positive role in clinical pharmacy services including medication order review, health product dispensing, storage and management of information about patients’ medical histories etc. [58, 59]. Clinical pharmacists need to keep abreast of these developments in order to position themselves optimally while maintaining their human relationships with healthcare teams and patients.

This study has some limitations. First, the instrument used to measure or evaluate the availability of facilities in secondary and tertiary hospitals and clinical pharmacists’ work performance was put into practice in large-scale research only after being verified by small samples from several tertiary hospitals in Jiangsu Province. But the data obtained from the large-scale research validated the instrument’s measuring effect. Second, since it seems impractical to randomly sample clinical pharmacists in the secondary and tertiary hospitals in China, convenience sampling was used in the multi-staged sampling procedure which may have led to a biased sample. Third, during the process of empowering weight to items of dependent variables, though the selected three principal components’ characteristic square root were all greater than 1, their cumulative variance contribution rate is 59.37%. Therefore, these three components may not fully represent the overall characteristics, resulting in a certain deviation between the weighted total score and the actual value.

## Conclusions

This study analysed the association of the availability of pharmaceutical facilities provided in Chinese hospitals and clinical pharmacists’ work performance. The results demonstrated that the availability of TCM pharmacy and drug researching room could significantly improve clinical pharmacists’ performance; while the availability of the preparation room might have certain negative impact on their work performance. These results may provide some evidence for hospitals to improve the performance of clinical pharmacists by increasing the availability of required facilities. The medical institutions may be supposed to design a specific ground for consultation of integrated use of Chinese and western medicine and purchase necessary equipment for decoction, in order to help pharmacists in TCM pharmacy provide high quality pharmaceutical service. Also, administrators of the hospitals are reminded to properly increase the investment in preparation room to expand its scale, standardize preparations’ production, so as to neutralize its negative impact on clinical pharmacists’ work performance.

### Supplementary Information

**Additional file 1.** Questionnaire.

## Data Availability

The datasets generated and/or analysed during the current study are not publicly available, because data used in this research is part of a larger data set exclusively constructed with the authors’ efforts, and this data set is to be used in the authors’ other researches, which required confidentiality. But they are available from the corresponding author on reasonable request.

## Abbreviation

TCM: Traditional Chinese Medicine

## Footnotes

1 x_1_: Emergency pharmacy, x_2_: Inpatient pharmacy, x_3_: Preparation room, x_4_: Laboratory of the control of drug, x_5_: Laboratory for research of drug, x_6_: Medicine store, x_7_: Clinical pharmacy, x_8_: Department of information, x_9_: Traditional Chinese medicine pharmacy, x_10_: Pharmacy of intravenous admixture service, x_11_: Level of hospitals, x_12_: Types of hospitals, x_13_: gender, x_14_: Marital status, x_15_: Technical title, x_16_: Educational background, x_17_: Age, x_18_: Years of practice.
