# Supplementary material for: Association of the availability of pharmaceutical facilities provided in secondary and tertiary hospitals with clinical pharmacists’ work performance
Source: BMC Health Serv Res. 2023 Dec 6;23:1361. doi: 10.1186/s12913-023-10390-1 (PMC10698899; doi:10.1186/s12913-023-10390-1)
Supplement: Supplementary file 1 — Additional file 1. Questionnaire. [file 12913_2023_10390_MOESM1_ESM.docx]

Additional file 1：Questionnaire

Questionnaire for the availability of pharmaceutical facilities and clinical pharmacists’ work performance

| Dear administrators of the hospital/clinical pharmacist:  This is a questionnaire about the availability of facilities required in pharmaceutical work as well as the clinical pharmacists’ work performance in China. It aims to study the impact of various facilities’ availability on the clinical pharmacists’ performance, so as to provide information and suggestions for the improving pharmaceutical working conditions to enhance clinical pharmacists’ performance. The survey results do not contain any of your identity information, and the survey results will be kept strictly confidential and used only for academic research and not for any commercial purpose.  The questions in the questionnaire are all single-choice questions or blank questions. Please answer according to your actual situation and attitude. If you have any questions about the requirements of the questionnaire, please feel free to ask the investigator. Thank you for your support and cooperation |
| --- |

| **Section 1** | |
| --- | --- |
| 1.What is your gender? | □Male □Female |
| 2. What is your age? _____years old | _____years old |
| 3. What is your current marriage situation? | □Unmarried □Married □Other (Divorced, Widowed, etc.) |
| 4. How many years have you worked in your current or similar position? _____years | _____years |
| 5. What is your technical title? (being awarded by passing corresponding exams organized by the state) | □Junior title □Intermediate title □Deputy senior title □senior title |
| 6. What is your degree and major at each level? | (Multiple choices) □Lower than Bachelor degree □Bachelor degree □Master degree □Doctoral degree |
| 7. What is the type of the hospital you work in | □ General hospital □Specialized hospital □Traditional Chinese Medicine Hospital □Other |
| 8. What is the level of the hospital you work in | □ secondary hospital □ tertiary hospital  □ Other |
| **Section 2 (to be filled by the administrators of the hospital)** | |
| Is the hospital you work in is availed with the following facilities? |  |
| 1. Outpatient pharmacy | □yes □no |
| 1. Emergency pharmacy | □yes □no |
| 1. Inpatient pharmacy | □yes □no |
| 1. Preparation room | □yes □no |
| 1. Laboratory of the control of drug | □yes □no |
| 1. Laboratory for research of drug | □yes □no |
| 1. Medicine store | □yes □no |
| 1. Clinical pharmacy | □yes □no |
| 1. Department of information | □yes □no |
| 1. Traditional Chinese medicine pharmacy | □yes □no |
| (11)Pharmacy of intravenous admixture service | □yes □no |
| **Section 3 You undertake the following responsibilities （to be filled by clinical pharmacists）** | |
| (1)Assessing the requirements of patients’ medication | □ strongly disagree □disagree □somewhat disagree □somewhat agree □agree □strongly agree |
| (2)Evaluating the rationality, safety, efficacy, economy, patients ‘compliance and potential problems of medication | □ strongly disagree □disagree □somewhat disagree □somewhat agree □agree □strongly agree |
| (3)Formulating and implementing health care plan | □ strongly disagree □disagree □somewhat disagree □somewhat agree □agree □strongly agree |
| (4)Conducting follow-up evaluation and drug monitoring | □ strongly disagree □disagree □somewhat disagree □somewhat agree □agree □strongly agree |
| (5)Recording information on patients’ medication | □ strongly disagree □disagree □somewhat disagree □somewhat agree □agree □strongly agree |
| (6)Checking patients’ medication history | □ strongly disagree □disagree □somewhat disagree □somewhat agree □agree □strongly agree |
| (7)Summarizing and assessing the problems of mediation | □ strongly disagree □disagree □somewhat disagree □somewhat agree □agree □strongly agree |
| (8)Optimizing mediation and improve the prognosis of patients | □ strongly disagree □disagree □somewhat disagree □somewhat agree □agree □strongly agree |
| (9)Collaborating with other health care providers in the medical team and performing your own unique responsibilities | □ strongly disagree □disagree □somewhat disagree □somewhat agree □agree □strongly agree |
| (10)Maintaining and improving professional competence | □ strongly disagree □disagree □somewhat disagree □somewhat agree □agree □strongly agree |
| (11)Scientific research and academic work | □ strongly disagree □disagree □somewhat disagree □somewhat agree □agree □strongly agree |
| (12)Teaching and guidance | □ strongly disagree □disagree □somewhat disagree □somewhat agree □agree □strongly agree |
